# Mitochondrial-derived peptides: emerging therapeutic targets at the intersection of mitochondrial dysfunction and pain

**DOI:** 10.3389/fpain.2026.2006909

**Published:** 2026-10-30

**Authors:** Zhe Wang, Jinglei Liao, Ping Lu, Yimin Xu, Jinhong Jiang

**Affiliations:** 1 School of Food and Biological Engineering, Xuzhou University of Technology, Xuzhou, China; 2 Jiangsu Province Key Laboratory of Anesthesiology and Brain Science, Jiangsu Province Key Laboratory of Anesthesiology, Xuzhou Medical University, Xuzhou, Jiangsu, China

**Keywords:** AMPK, humanin, mitochondrial biogenesis, mitochondrial-derived peptides, MOTS-c, neuropathic pain, SHLPs

## Abstract

Mitochondrial-derived peptides (MDPs), including humanin and MOTS-c, are endogenous peptide signaling molecules that regulate cellular stress responses and metabolic homeostasis. Mitochondrial dysfunction is a convergent downstream mechanism across diverse chronic pain states, yet existing therapies target only limited neural mechanisms. Recent preclinical evidences demonstrate that humanin and MOTS-c exert analgesic effects across multiple pain models through shared mechanisms converging on AMPK-PGC-1α-mediated mitochondrial biogenesis and suppression of oxidative stress and neuroinflammation. Humanin attenuates metabolic, toxic, and traumatic neuropathic pain while reducing pain-related markers in dorsal root ganglia. MOTS-c shows broader efficacy across neuropathic, diabetic, inflammatory, and cancer-induced bone pain models, with mechanistic evidence linking its effects to AMPK activation, microglial inhibition, and restoration of mitochondrial function. Endogenous MDP levels decline in pain states, suggesting that their loss contributes to pain pathogenesis and that restoration may be therapeutic. SHLPs, particularly SHLP2, have not been directly studied in pain but exhibit anti-inflammatory and anti-apoptotic properties that provide a plausible indirect link. This review synthesizes current evidence on MDPs in pain, and proposes priorities for translating these endogenous peptides into therapeutic strategies for chronic pain.

## Introduction

Chronic pain, particularly neuropathic pain, remains a major therapeutic challenge. Existing drugs, including opioids, gabapentinoids, tricyclic antidepressants, and serotonin-norepinephrine reuptake inhibitors, target a limited set of neural targets and produce meaningful relief in only a subset of patients (1–3). Moreover, these agents are often accompanied by dose-limiting side effects such as dizziness, drowsiness, and weight changes. This limited efficacy reflects the complex, multifactorial pathophysiology of chronic pain, which involves maladaptive changes in peripheral nociceptors, spinal dorsal horn circuits, and supraspinal processing centers that collectively maintain the pain state (4–6). Yet these seemingly disparate maladaptive changes may share a deeper cellular basis-mitochondrial dysfunction (7, 8).

**Figure 1:**
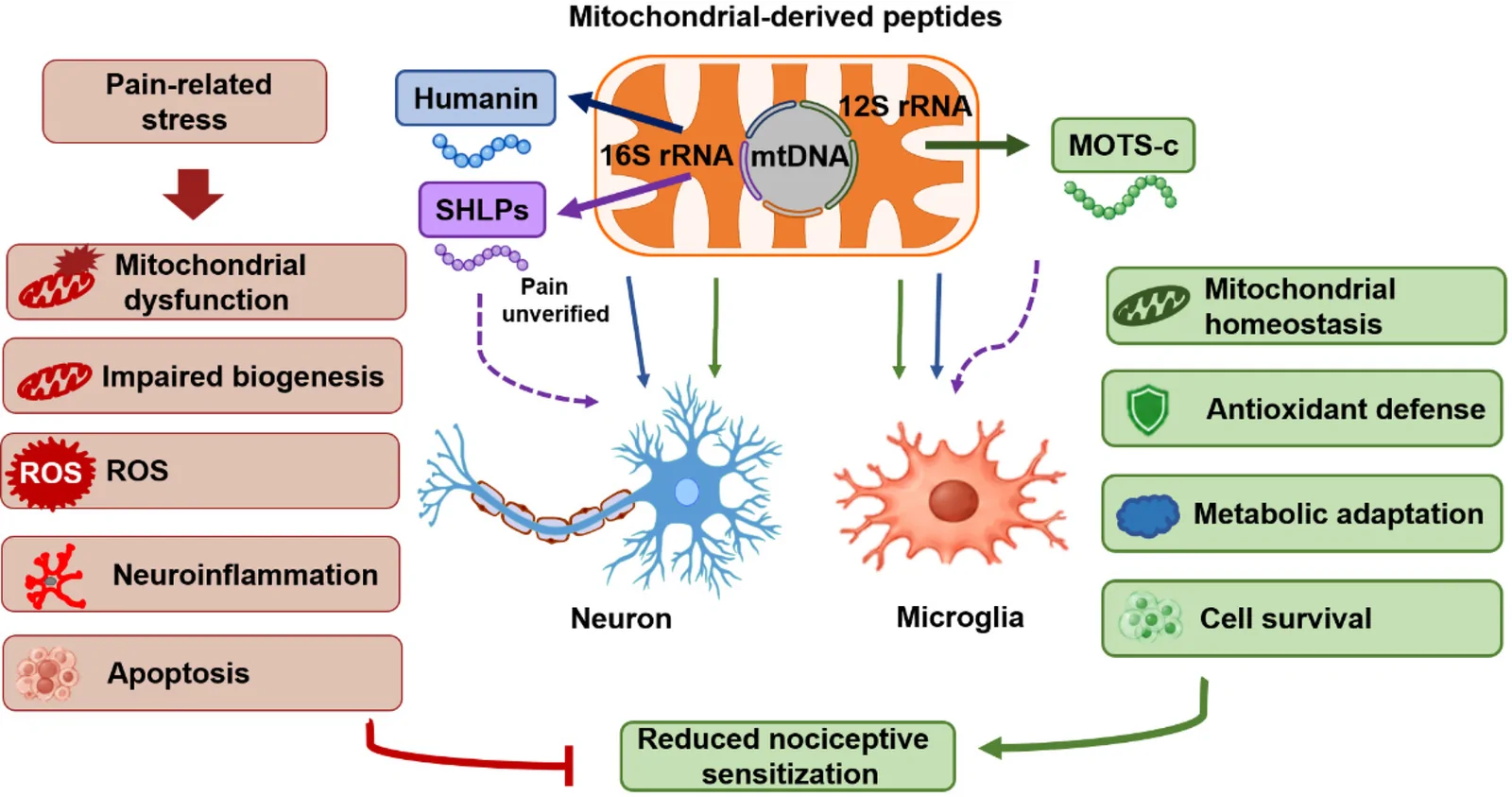
Sources and mechanisms of action of MDPs. Dashed lines explicitly indicate that the role of SHLPs in pain has not yet been directly validated.

**Figure 2:**
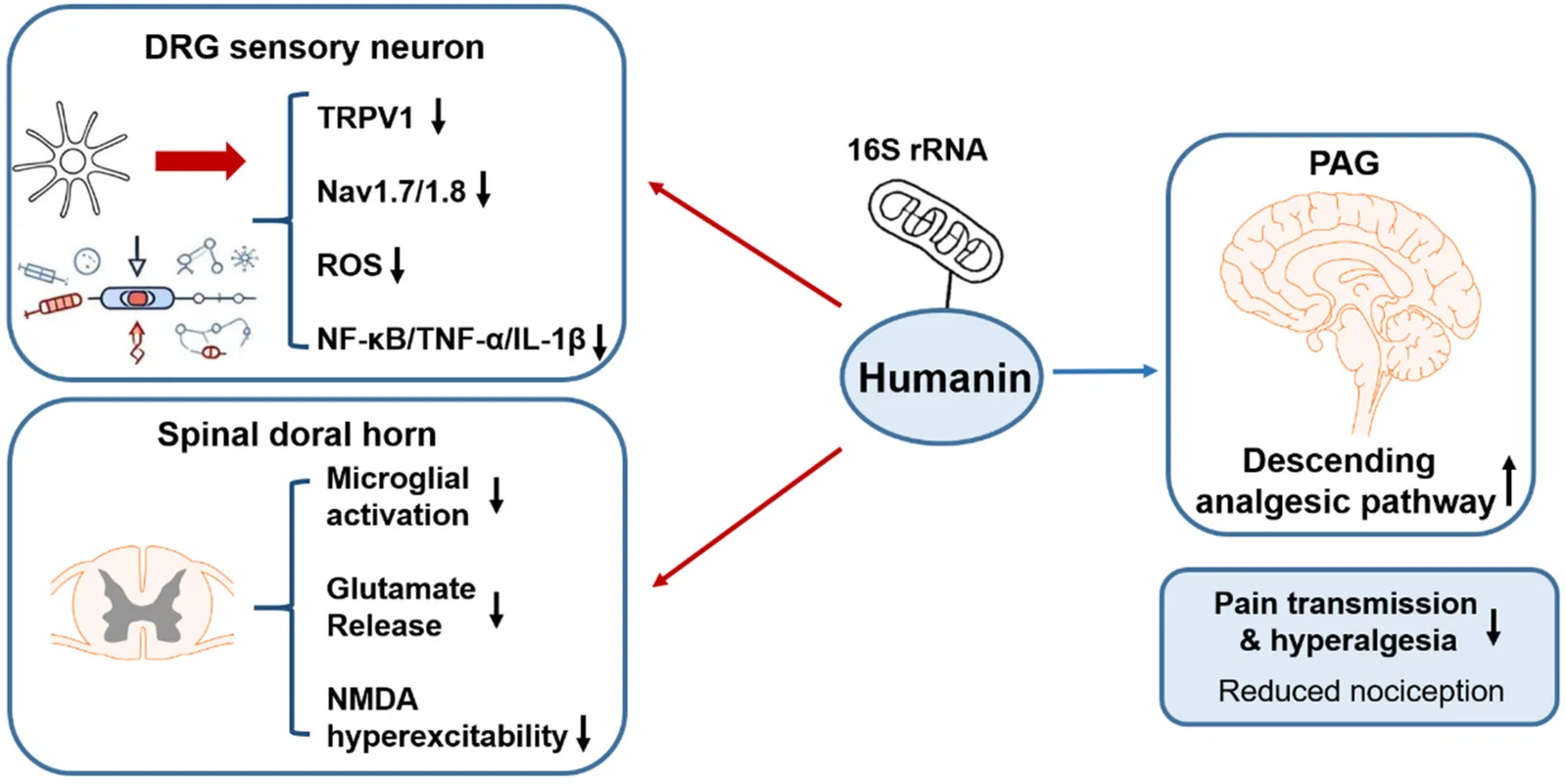
Humanin-mediated pain modulation. Blue arrows indicate activation; red arrows indicate inhibition.

**Figure 3:**
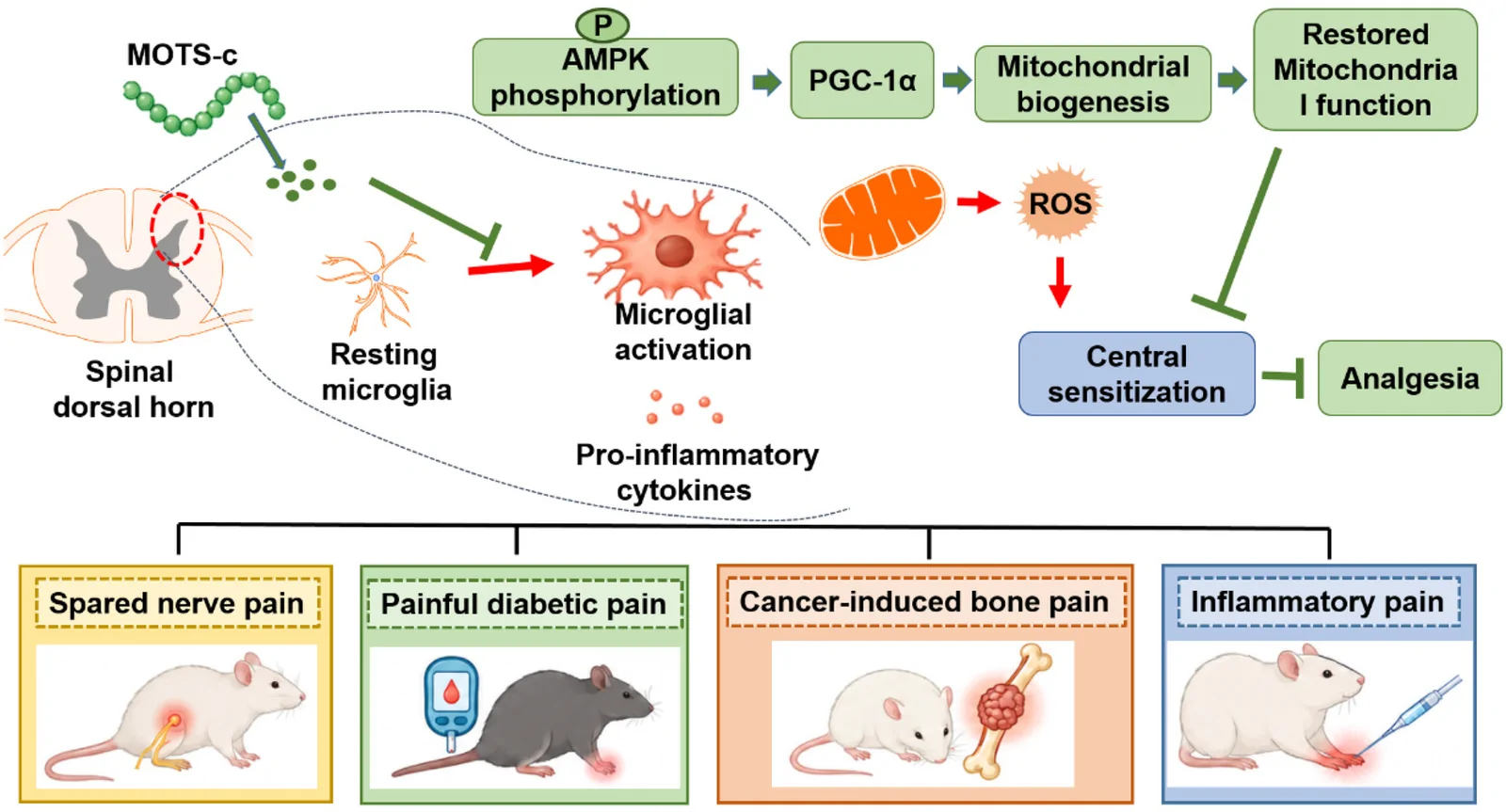
Broad-spectrum analgesic mechanisms of MOTS-c. Highlight the AMPK-PGC-1α-mitochondrial biogenesis axis, microglial inhibition, and central sensitization.

Mitochondrial dysfunction has emerged as a convergent downstream mechanism linking diverse pain etiologies (7–9). Whether pain originates from metabolic derangements (diabetic neuropathy), neurotoxic insults (chemotherapy-induced neuropathy), traumatic nerve injury, or inflammatory conditions, a common pathological signature is observed: excessive mitochondrial reactive oxygen species (ROS) production, impaired mitochondrial biogenesis, disrupted mitochondrial dynamics, and consequent activation of neuroinflammatory signaling (9–11). This convergence has therapeutic implications: strategies that restore mitochondrial homeostasis may provide benefits across pain types that are otherwise mechanistically distinct.

Recently, mitochondrial-derived peptides (MDPs) represent a unique class of endogenous regulators of mitochondrial function (12). Unlike conventional mitochondrial-targeted antioxidants such as MitoQ, which scavenge ROS passively, MDPs act as peptide signaling molecules that actively reprogram cellular metabolism and stress responses (12, 13). As shown in Figure 1, three MDPs have received particular attention: (1) humanin, a 24-amino-acid peptide encoded by the mitochondrial 16S rRNA region (14); (2) MOTS-c (mitochondrial open reading frame of the 12S rRNA type-c), a 16-amino-acid peptide with a highly conserved N-terminal sequence across species (15); and (3) SHLPs (small humanin-like peptides), a group of small peptides encoded by the mitochondrial 16S rRNA region, comprising six members (SHLP1-SHLP6) (16). These MDPs exert pleiotropic effects through enhancing mitochondrial biogenesis while suppressing oxidative stress and inflammation (17).

The relevance of MDPs to pain has only recently been explored, but the findings are compelling. Humanin attenuates neuropathic pain across metabolic, toxic, and traumatic models while reducing pain-related markers in dorsal root ganglia (18). MOTS-c demonstrates efficacy in spared nerve injury, painful diabetic neuropathy, inflammatory pain, and cancer-induced bone pain, with mechanistic evidence implicating AMPK-mediated mitochondrial biogenesis and microglial inhibition (19–22). Although SHLPs have not yet been directly reported to be associated with pain, SHLP2 has been shown to possess anti-inflammatory and anti-apoptotic properties, reducing ROS production and downregulating apoptotic markers such as p53 and BAX (16, 23). Given that neuroinflammation and apoptosis are important mechanisms underlying the maintenance of neuropathic pain (24, 25), these protective effects of SHLP2 suggest its potential indirect involvement in pain regulation, warranting further validation in pain models in the future. Notably, endogenous MDP levels decline in pain states (19, 22), suggesting that their loss may contribute to pain pathogenesis and that restoration could be therapeutic.

This review synthesizes current preclinical evidence on MDPs in pain, identifies shared mechanistic nodes, and critically evaluates the limitations and translational priorities for this emerging field.

## Humanin: neuroprotection and analgesia across neuropathic pain conditions

Humanin, a 24-amino acid peptide encoded by the mitochondrial 16S rRNA region (MT-RNR2 gene), is the pioneering mitochondrial-derived peptide. It was initially characterized as a neuroprotective agent in Alzheimer's disease models, where it suppresses amyloid-β-induced toxicity through PI3 K/AKT signaling (14, 26). Subsequent investigations revealed its presence not only in the brain but also in peripheral tissues including the kidney, skeletal muscle, heart, testis, and liver (26). Consistent with this widespread distribution, humanin confers protection in diverse disease models by attenuating mitochondrial dysfunction, ROS production, and inflammatory cytokine levels (27, 28).

A recent study evaluating humanin in three mechanistically distinct neuropathic pain models: streptozotocin (STZ)-induced diabetic neuropathy, oxaliplatin-induced chemotherapy neuropathy, and sciatic nerve cuffing-induced traumatic neuropathy (18). Humanin (4 mg/kg, intraperitoneal) administered over 15 days significantly increased pain thresholds in all three models, assessed by hot plate, cold plate, and von Frey tests. Mechanistic studies showed that in diabetic mice, chronic humanin treatment reduced elevated expression of pain-related markers in dorsal root ganglia (DRG), including the NMDA receptor, substance P, TRPV1, and interleukin-1β (IL-1β) (29). These markers represent distinct nodes in nociceptive signaling: NMDA receptors mediate central sensitization, substance *P* is a key nociceptive neurotransmitter, TRPV1 transduces thermal and inflammatory stimuli, and IL-1β drives neuroinflammation (30–32). Humanin's ability to reduce all four simultaneously suggests broad suppression of nociceptive sensitization rather than targeting a single pathway. At the systemic level, humanin administration in neuropathic pain models increased antioxidant enzyme levels and anti-inflammatory cytokines while reducing oxidative stress markers and pro-inflammatory cytokines in serum (18, 29). This systemic anti-inflammatory and antioxidant profile complements the local effects in DRG and likely contributes to the observed analgesia.

A critical unanswered question is whether humanin's analgesic effects are independent of its metabolic effects. As shown in Figure 2, humanin improves insulin sensitivity and glucose metabolism in diabetic models (33), and it is possible that improved glycemic control indirectly reduces neuropathic pain. However, humanin's efficacy in non-diabetic pain models, such as oxaliplatin-induced and traumatic cuffing-induced neuropathy, argues against a purely metabolic mechanism (18). Future studies using metabolically neutral pain models or tissue-specific humanin receptor deletion would help resolve this question. Another significant gap concerns the role of glial cells. Current mechanistic evidence focuses almost exclusively on neurons, yet microglia and astrocytes are established drivers of neuropathic pain through release of pro-inflammatory mediators (34, 35). Whether humanin modulates glial activation directly or indirectly through neuronal signaling remains unknown. Given that humanin reduces IL-1β, a cytokine produced predominantly by activated microglia (34), glial mechanisms are likely involved but have not been formally tested.

## MOTS-c: broad-spectrum analgesia through AMPK-mediated mitochondrial biogenesis

MOTS-c is a 16-amino acid MDP encoded by the 12S rRNA region of mitochondrial DNA (15). Under resting conditions, it is primarily localized to mitochondria, but under metabolic stress it translocates to the nucleus in an AMPK-dependent manner, regulating nuclear gene expression to maintain cellular homeostasis (36). MOTS-c has gained attention as a metabolic regulator with therapeutic potential in obesity, insulin resistance, and inflammatory diseases (37, 38). Unlike Humanin, MOTS-c has more direct evidence in pain research. Multiple studies have shown that MOTS-c exerts analgesic effects across various pain models, with mechanisms involving AMPK-mediated mitochondrial biogenesis and inhibition of spinal microglial activation, thereby alleviating neuroinflammation and neuronal oxidative damage (19–22).

The first direct evidence for MOTS-c in pain came from a spared nerve injury (SNI) model, in which MOTS-c levels were significantly decreased in plasma and spinal dorsal horn (19), suggesting that endogenous MOTS-c depletion may contribute to pain pathogenesis. Intrathecal MOTS-c produced dose-dependent antinociception, mechanistically linked to inhibition of spinal microglial activation and reduced pro-inflammatory cytokine production. Subsequent work extended MOTS-c's efficacy to painful diabetic neuropathy (PDN) (22). In STZ-induced PDN mice, MOTS-c administered via intrathecal, intraperitoneal, or intraplantar routes significantly improved mechanical allodynia and thermal hyperalgesia. Mechanistically, MOTS-c enhanced AMPKα1/2 phosphorylation and PGC-1α expression in the lumbar spinal cord, accompanied by restored mitochondrial biogenesis, inhibited microglial activation, and decreased pro-inflammatory factor production (22). MOTS-c has also shown efficacy in cancer-induced bone pain (BCP), alleviating pain through AMPK-mediated mitochondrial biogenesis (21). Beyond these models, it demonstrated anti-inflammatory and analgesic effects in the formalin test, a model of acute inflammatory pain, attributed to both central and peripheral anti-inflammatory actions (20).

As shown in Figure 3, the breadth of MOTS-c's efficacy across neuropathic, diabetic, inflammatory, and cancer-related pain is striking and distinguishes it from humanin, for which evidence is currently limited to neuropathic pain models. This breadth likely reflects the central role of AMPK-PGC-1α signaling in cellular stress responses: by activating this pathway, MOTS-c enhances mitochondrial function and reduces oxidative stress across diverse cell types and pathological contexts (19). An important translational development is the clinical evaluation of CB4211, a MOTS-c analog, in a Phase 1a/1b clinical trial for nonalcoholic steatohepatitis (NASH) and obesity (NCT03998514). CB4211 was well-tolerated and showed encouraging improvements in liver enzymes and glucose levels after four weeks of treatment. While this trial was not designed for pain endpoints, it establishes that MOTS-c-based therapeutics can be safely administered to humans, providing a foundation for future pain-focused clinical studies.

## SHLPs: anti-inflammatory and anti-apoptotic properties suggest potential indirect pain regulation

SHLPs (small Humanin-like peptides, 1–6) are six small peptides encoded by the same MT-RNR2 gene, sharing a mitochondrial origin with humanin (16). However, unlike humanin and MOTS-c, SHLPs have no direct reports in pain models to date. Current evidence is limited to other disease systems, among which data on SHLP2 are the most prominent. The anti-inflammatory and anti-apoptotic properties of SHLP2 have been repeatedly validated in various non-pain models. In an oxidative stress-induced osteoblast precursor injury model, SHLP2 (10 µM) significantly reduced ROS production, downregulated pro-apoptotic markers such as p53 and BAX, and elevated anti-apoptotic BCL-2 levels (23). In a cellular model of age-related macular degeneration (AMD), SHLP2 protected retinal pigment epithelial cells from amyloid-β toxicity by inhibiting apoptotic pathways and upregulated mitochondrial respiratory chain complex I expression by up to 350% (39). Furthermore, in an allergic asthma model, SHLP2 attenuated airway inflammation by improving mitochondrial function and inhibiting ETV5/GSDMD-mediated pyroptosis (40).

The mechanistic commonality underlying these protective effects lies in maintenance of mitochondrial function and suppression of cell death pathways. SHLP2 has been shown to activate ERK and STAT3 survival signaling pathways, while also participating in energy homeostasis regulation through binding to the CXCR7 receptor (16). Regarding potential relevance to pain, neuroinflammation and apoptosis are important mechanisms underlying the maintenance of neuropathic pain (41, 42). Studies have shown that apoptotic cells are present at the injury site, in distal dorsal root ganglia, the spinal cord, and some brain regions, and that inefficient clearance of apoptotic cells is associated with pain persistence (42). In a CCI rat model, 14 days after injury (when pain has become chronic), cells in laminae I-III of the spinal dorsal horn decreased by approximately 23%, TUNEL-positive nuclei appeared, and the pro-apoptotic gene bax was upregulated by approximately 40% (41). This suggests that in chronic pain states, apoptosis-related genes remain persistently active, potentially providing a cellular basis for the maintenance of pain.

Unlike Humanin and MOTS-c, which already have established evidence in pain models, SHLPs remain a blank area in pain research. The anti-inflammatory and anti-apoptotic properties of SHLP2 therefore constitute a plausible but as-yet-unverified indirect link. Notably, the functions of SHLP family members are not uniform: SHLP3 exerts cytoprotective effects and modulates inflammatory markers, SHLP4 promotes cell proliferation, whereas SHLP6 exhibits pro-apoptotic effects, opposite in direction to SHLP2/SHLP3 (16, 43). The existing protective data on SHLP2 provide a mechanistic basis for future validation in pain models.

## Common and distinct mechanisms of MDPs

The core function of MDPs is to mediate mito-nuclear crosstalk, relaying mitochondrial stress signals to the nuclear genome and thereby coordinating antioxidant defense, metabolic reprogramming, and cell survival. However, humanin, MOTS-c, and SHLPs occupy distinct positions within this framework. Humanin is biased toward cytoprotection and apoptosis inhibition, acting primarily through PI3 K/AKT and JAK2/STAT3 pathways, and in some contexts it more closely resembles a “cytokine-like” extracellular signaling molecule (16). MOTS-c, by contrast, is biased toward metabolic adaptation and mitochondrial quality control; under metabolic stress, it translocates to the nucleus, regulates nuclear gene expression, and thereby activates AMPK-dependent metabolic adaptation programs (36). Functional divergence is most pronounced among the SHLPs: SHLP2 and SHLP3 resemble humanin in suppressing apoptosis, reducing ROS, and improving mitochondrial metabolism (39), whereas SHLP6 exhibits opposing pro-apoptotic activity and inhibits tumor growth through VEGF-A downregulation. This coexistence of “protective” and “pro-apoptotic” functions makes SHLPs a unique model for studying the functional plasticity of MDPs (44, 45). No direct evidence for SHLPs in pain models currently exists, but the mitochondrial protective and central neuron-activating properties of SHLP2 suggest potential analgesic relevance.

## Endogenous MDP level changes: mechanisms and biomarker potential

Although MDPs are increasingly recognized as endogenous regulators of mitochondrial function, the mechanisms underlying changes in their expression, processing, and release under pathological conditions remain poorly understood. Because MDPs are encoded by mitochondrial DNA, their production depends on mitochondrial genomic integrity, transcription, and translation. Humanin and SHLPs are derived from the MT-RNR2 gene within the 16S rRNA region, whereas MOTS-c is encoded by the 12S rRNA region (15–17). Therefore, impaired mtDNA stability, mitochondrial transcription, or mitochondrial translation would be expected to reduce MDP expression. Mitochondrial dysfunction is a shared feature of chronic pain states and includes excessive ROS production, impaired mitochondrial biogenesis, disrupted mitochondrial dynamics, mitochondrial calcium overload, and reduced mtDNA copy number (7–11). These abnormalities may directly affect MDP production, as oxidative stress can damage mtDNA and mitochondrial RNA, impair mitochondrial ribosome function, and disrupt post-translational processing of MDP precursors.

The release of MDPs may also be affected by pathological conditions. Under metabolic stress, MOTS-c translocates to the nucleus in an AMPK-dependent manner (36), suggesting that MDP localization is dynamically regulated. Mitochondrial stress, membrane permeabilization, and extracellular vesicle-mediated secretion may further influence the extracellular availability of MDPs. Together, these observations suggest a vicious cycle in which pain-related mitochondrial dysfunction reduces endogenous MDP production, which in turn weakens mitochondrial protection, amplifies oxidative stress and neuroinflammation, and reinforces nociceptive sensitization. Consistent with this framework, a key observation in the SNI model is that MOTS-c is significantly decreased in both plasma and the spinal dorsal horn 7 days after injury (19). The pain state itself appears to suppress MDP synthesis or secretion, and the resulting reduction in endogenous MDPs in turn weakens mitochondrial protection, forming a vicious cycle.

This finding suggests that MDPs may play a dual role: they may serve both as “depletable protective factors” under pain conditions and as endogenous biomarkers reflecting mitochondrial reserve status. Notably, patterns of MDP level changes have been systematically studied in multiple diseases. In Alzheimer's disease, for example, transcript levels of humanin and MOTS-c in blood and plasma are significantly lower than in controls (46). Another study found decreased plasma MOTS-c levels in AD patients, whereas humanin levels were reduced in AD but unchanged in type 2 diabetes, suggesting that MDP changes are disease-specific (47). In the cardiovascular field, circulating humanin levels are significantly reduced in patients with coronary artery disease and myocardial infarction, and low humanin is an independent risk factor for myocardial infarction (48); MOTS-c is similarly reduced in patients with stable coronary artery disease and can serve as an independent predictor of coronary lesions, while both MOTS-c and PGC-1α levels decline in acute coronary syndrome (49, 50). In metabolic diseases, circulating MOTS-c levels are significantly reduced in obese male children and adolescents and correlate with insulin resistance (51), and serum humanin levels are also significantly lower in women with gestational diabetes mellitus than in controls (52). These observations raise the possibility that MDPs are not merely downstream markers of mitochondrial dysfunction.

An unexplored but translationally valuable question is whether circulating MDP levels correlate with the stage of pain chronification or pain severity. Acute endurance exercise has been shown to significantly increase circulating humanin levels, with MOTS-c also showing an upward trend (53). Whether exercise-induced analgesia is partially mediated by MDPs is a hypothesis worth testing in animal models (54, 55). If confirmed, MDPs could become a molecular node linking the “exercise-mitochondria-analgesia” axis.

## Translational prospects: positioning of MDPs, mitochondrial transplantation, and mitochondrial combination therapy in pain

To date, no MDP-based therapeutic has been clinically evaluated with pain as a primary endpoint. Current evidence for analgesic effects of MDPs is therefore entirely preclinical. Compounds with direct preclinical evidence of analgesic effects include MOTS-c and Humanin. MOTS-c has shown analgesic effects across neuropathic, diabetic, inflammatory, and cancer-induced bone pain models, with mechanisms involving AMPK-PGC-1α-mediated mitochondrial biogenesis and microglial inhibition (19–22). Humanin has demonstrated efficacy in metabolic, toxic, and traumatic neuropathic pain models, accompanied by reduced pain-related markers in DRG (18, 29).

By contrast, several compounds with potential relevance to pain are primarily supported by their effects on mitochondrial function or metabolic regulation rather than by direct analgesic evidence. CB4211, a MOTS-c analog, has completed evaluation in a Phase 1a/1b trial for NASH and obesity (NCT03998514). CB4211 was well-tolerated, and after four weeks of treatment, liver enzymes (ALT, AST) and blood glucose levels were significantly reduced, with measurable reductions in liver fat as well. Although this trial was not designed with pain endpoints, it established that MDP-based therapeutics can be safely administered to humans, providing a foundation for future pain-focused clinical studies. Because CB4211's relevance to pain is inferred from its mitochondrial and metabolic effects rather than from direct analgesic evidence, it should be classified as a compound with indirect pain relevance.

Meanwhile, mitochondrial-targeted strategies in the pain field are advancing along a different path. A 2026 study published in Nature by Ji Ru-Rong's group at Duke University revealed a previously overlooked mechanism: satellite glial cells (SGCs) in the DRG transfer mitochondria to sensory neurons via MYO10-driven tunneling nanotubes (TNTs) (56). In diabetic and chemotherapy-induced neuropathic pain models, this transfer process is impaired, with the nanogap between SGCs and neurons expanding from approximately 0.1 µm to 1–4 µm, interrupting mitochondrial supply. Transplanting healthy donor SGCs or their purified mitochondria into the DRG of diabetic mice significantly improved hyperalgesia and promoted intraepidermal nerve fiber regeneration. This finding aligns with the logic of MDPs, both aim to restore mitochondrial function, but differs in approach: MDPs “pharmacologically activate endogenous mitochondrial repair programs,” whereas mitochondrial transplantation “exogenously replaces damaged organelles.”

An intermediate path closer to clinical application is being tested. A Phase 2 clinical trial (Mito-T2D_PPN study, registration number ACTRN12624000623594) is evaluating a ubiquinone-based combination therapy (coenzyme Q10 + magnesium + α-lipoic acid + thiamine) for type 2 diabetes-related painful peripheral neuropathy (57). This trial directly targets pain and provides a proof-of-concept opportunity for mitochondria-targeted therapies. If this combination therapy shows benefit on pain endpoints, it would provide proof of concept for the clinical development of MDPs. After all, MDPs are mechanistically more targeted than exogenous supplements, as they directly activate the AMPK-PGC-1α signaling axis rather than passively supplying substrates.

Several priorities should guide future pain-focused clinical development of MDPs. First, clinical trials should incorporate validated pain endpoints. Second, circulating MDP levels should be measured as potential biomarkers. Third, studies should distinguish direct analgesic effects from indirect benefits of improved metabolic control. Fourth, combination strategies with existing analgesics should be explored.

Taken together, the unique advantage of MDPs lies in their “endogenous” nature: they are signaling molecules that the body itself uses to maintain mitochondrial homeostasis, theoretically offering better signal integration capacity and lower immunogenicity risk. The translational prospects of MDPs in pain depend on answering two key questions: First, is the decline in MDP levels a driving factor or an accompanying phenomenon of pain? Second, is the analgesic effect of MDPs a direct restoration of mitochondrial function, or an indirect result of downstream anti-inflammatory/anti-apoptotic pathways? Clarifying these two questions will lay the foundation for MDPs’ transition from “promising preclinical targets” to “testable clinical candidates.”

## Conclusion

MDPs represent an emerging class of endogenous regulatory molecules that converge on a shared pathological node in chronic pain, mitochondrial dysfunction. Although humanin, MOTS-c, and SHLP2 differ in sequence, tissue distribution, and signaling preferences, all possess the capacity to attenuate oxidative stress, suppress neuroinflammation, and restore mitochondrial homeostasis. Among them, MOTS-c currently has the most direct and broadest evidence in the pain field, spanning neuropathic pain, diabetic pain, inflammatory pain, and cancer-induced bone pain, with its mechanism centered on AMPK-PGC-1α-mediated mitochondrial biogenesis and microglial inhibition. Humanin has demonstrated efficacy across multiple mechanistically distinct neuropathic pain models, but its glial mechanisms and whether its analgesic effects are independent of metabolic actions remain unclear. SHLPs, particularly SHLP2, remain a blank area in pain research; their anti-inflammatory and anti-apoptotic properties provide a plausible but as-yet-unverified indirect link, and the functional heterogeneity within the family, with SHLP2/SHLP3 and SHLP6 acting in opposite directions, suggests that SHLPs cannot be viewed as a unified target.
